# Restless legs syndrome and functional limitations among American elders in the Health and Retirement Study

**DOI:** 10.1186/1471-2318-12-39

**Published:** 2012-07-26

**Authors:** Dominic J Cirillo, Robert B Wallace

**Affiliations:** 1 Department of Internal Medicine, University of Iowa Carver College of Medicine, Iowa City, IA, USA; 2 Department of Epidemiology, University of Iowa College of Public Health, Iowa City, IA, USA

## Abstract

**Background:**

Restless legs syndrome (RLS) is a common condition associated with decreased quality of life in older adults. This study estimates the prevalence, risk factors, and functional correlates of among U.S. elders.

**Methods:**

Subjects (n = 1,008) were sub-sampled from the 2002 cross-sectional interview survey of the Health and Retirement Study (HRS), a nationally representative study of U.S. elders. Symptoms and sleep disturbances consistent with RLS were identified. Activities of daily living (ADL), instrumental activities of daily living (IADL), and limitations for mobility, large muscle groups, gross and fine motor function were measured using standardized questions. Incident functional limitations were detected over six years of observation.

**Results:**

The prevalence of RLS among U.S. elders born before 1947 was 10.6%. Factors associated with increased prevalence RLS at baseline included: overweight body mass index (multivariate adjusted prevalence ratio = 1.77; 95% confidence interval (CI) 1.05-2.99); mild-to-moderate pain (2.67, 1.47-4.84) or pain inferring with activity (3.44, 2.00-5.93); three or more chronic medications (2.54, 1.26-5.12), highest quartile of out-of-pocket medical expenses (2.12, 1.17-3.86), frequent falls (2.63, 1.49-4.66), health limiting ability to work (2.91, 1.75-4.85), or problems with early waking or frequent wakening (1.69, 1.09-2.62 and 1.55, 1.00-2.41, respectively). Current alcohol consumption (0.59, 0.37-0.92) and frequent healthcare provider visits (0.49, 0.27-0.90) were associated with decreased RLS prevalence. RLS did not predict incident disability for aggregate measures but was associated with increased risk for specific limitations, including: difficulty climbing several stair flights (multivariate-adjusted hazard ratio = 2.38, 95% CI 1.39-4.06), prolonged sitting (2.17, 1.25-3.75), rising from a chair (2.54, 1.62-3.99), stooping (2.66, 1.71-4.15), moving heavy objects (1.79, 1.08-2.99), carrying ten pounds (1.61, 1.05-2.97), raising arms (1.76, 1.05-2.97), or picking up a dime (1.97, 1.12-3.46).

**Conclusions:**

RLS sufferers are more likely to have functional disability, even after adjusting for health status and pain syndrome correlates.

## Background

Restless Legs Syndrome (RLS) is characterized by unpleasant sensations of tingling, creeping, bubbling, or “tunneling bugs” [1]. The International RLS Study Group established diagnostic criteria for RLS, including: a strong urge to move, predominantly in the legs; relief with movement, stretching, or rubbing; and symptomatic worsening at night or with inactivity [2]. RLS is a common chronic condition, with prevalence estimates ranging from 5-10% [3,4], with some geographic variability [3,5-7]. Many RLS cases are secondary to other causes, such as iron deficiency, pregnancy, and neurologic, rheumatologic, or renal diseases, but more than 60% of cases are idiopathic [8,9]. Primary RLS prevalence in the U.S. is likely 1-3% [9], and is likely more common in women and during the seventh and eighth decades [5]. RLS is clinically diagnosed but can be confirmed by polysomnography or the Suggested Immobilization Test [8], and no confirmatory laboratory tests exist [10]. Many RLS sufferers seek care but the average time to diagnosis is two years [11], and the RLS Epidemiology, Symptoms, and Treatment (REST) study confirmed that many are misdiagnosed and given inappropriate treatments [3].

RLS also impacts health-related quality of life [12-14]. Like those with other chronic conditions, RLS sufferers have lower scores on the Medical Outcome Study Short Form 36 (SF-36) in both clinical [15] and population-based samples [5,12]. RLS sufferers may be less productive at work [9]. RLS sufferers report negative influences on mood, energy, and daily activities [5], and are shown to have more daytime fatigue, work difficulties, and driving impairment [4]. Among elders, severe RLS has been associated with poorer social function, daily function, sleep quality, and emotional well-being [16]. However, the full impact RLS has on disability has yet to be shown.

The Health and Retirement Study (HRS) is a nationally representative cohort of economic and physical health of elders [17]. Since 1992, HRS has provided comprehensive and detailed information on many domains, including health status, employment, disability, and net worth of U.S. elders. A sub-sample was queried about RLS symptoms in 2002, providing an opportunity to report epidemiologic correlates of RLS and, more importantly, longitudinal associations between RLS and subsequent functional limitations.

## Methods

The Health and Retirement Study is an ongoing cohort study consisting of a representative sample of over 20,000 Americans born prior to 1948, with interview data collected biennially on demographics, health status, employment, income and wealth, and insurance; full details are described elsewhere [17]. In 2002, the overall response rate of the cohort was 86.9% [17]. Sub-samples (“modules”) are asked additional questions on ancillary topics after the main survey [18]. The module topic is revealed after respondents consent. Of 1,502 randomly selected respondents, 1,058 (70.4%) agreed to complete the 2002 RLS module. Nursing home residents and subjects outside the eligible age-range for HRS (e.g. spouses of age-eligible respondents) were excluded from the analysis (n = 50), yielding a final sample size of 1,008. HRS was reviewed by the University of Michigan Institutional Review Board [17].

The 2002 RLS module predated validated instruments for measuring RLS outside of a clinical setting [2]. Respondents reported presence of resting symptoms, including “crawling, tingling or achy sensations in your arms or legs”, feeling “restless, fidgety, or unable to sit still”, “feel the need to move your legs, rub your legs, or stretch your legs”, “you or bedpartner noticed twitching or kicking of your arms and legs”, or “itching sensations anywhere on your body.” Respondents reported how frequently they experienced “unpleasant feelings” in the legs – “for example, creepy-crawling or tingly feelings—that make you feel restless and keep you from getting a good night’s sleep”, with options ranging from “never” to “every night”. Subjects were also asked how frequently they “get pain or cramps in [the] legs to the point where it is uncomfortable and disturbs sleep”. Symptomatic individuals were asked if they sought medical advice, whether treatment was offered, and whether treatment helped.

Potential health correlates were derived from the parent HRS study, and full questionnaires are available at http://hrsonline.isr.umich.edu[17]. Subjects self-reported demographic information, including race, gender, date of birth, and educational attainment, upon study entry. Health status is evaluated with each interview wave, including medication use, chronic medical conditions (hypertension, diabetes, heart disease, stroke, lung disease, psychiatric disease, arthritis, and cancer), health behaviors, and symptoms, including general sleep concerns and pain. Bodily pain experience was categorized into three levels: severe (pain makes normal activities difficult), mild-moderate (troubled by pain but does not limit activity), or no troubling pain. Pain site was not localized. General sleep-related concerns include difficulty falling asleep, waking frequently at night, and waking early and unable to return to sleep. Depressive symptoms were assessed using a shortened version of the Center for Epidemiologic Studies Depression (CES-D) Scale [19], with depressive symptoms noted for scores of two or more (corresponding to the 20^th^ most depressed percentile).

Smoking status was current, former, or never. Alcohol use was classified as current (yes/no), with additional quantification of drinking days per week and drinks per drinking day. Body mass index (BMI) was calculated using reported weight and height, and categorized as BMI < 25, 25 ≤ BMI < 30, or BMI ≥ 30 (18 missing values were classified at the median, 26.6). Self-reported health status was classified as “Excellent”, “Very Good”, “Good”, “Fair”, or “Poor”. For this analysis, the top two and the bottom two response categories were collapsed. Self-reported vigorous activity at baseline was defined as at least three vigorous activity sessions per week. In subsequent waves, frequency of physical activity was further broken into episodes of mild, moderate, or vigorous activity per week. Individuals reported paid employment outside the home and whether personal health limited their ability to work. Income was calculated from all sources, and divided into quartiles. Total medical expenses and out-of-pocket medication expenses were collected, imputed as necessary from the RAND datafiles, and divided into quartiles. Subjects self-reported falls, hospitalizations, and frequency of healthcare provider visits in the previous two years. For the analysis, falls were grouped as “None”, “One”, or “Two or more”. Frequency of provider visits were grouped as “0-3”, “4-7”, “8-11”, or “12 or more”. Self-reported medications for each of the above chronic conditions were queried, and for this analysis were categorized as “None”, “One”, “Two”, or “Three or more”.

Functional status was assessed using several standardized instruments. Variables were dichotomized to “at least some difficulty” versus no difficulty. Activities of daily living (ADL) assessed any difficulties on five tasks: bathing, eating, dressing, toileting, and transferring. Instrumental activities of daily living (IADL) included difficulties with: managing money, managing medications, preparing meals, going shopping, or using a telephone. Mobility limitations included limitations on walking and climbing stairs. Additional motor limitations were grouped based on *a priori* definitions. Gross motor limitations included any difficulty walking one block, climbing one flight of stairs, transferring, bathing, or requiring a walking assist device. Fine motor limitations included difficulty picking up a dime, feeding oneself, or dressing. Large muscle group limitations included sitting for two hours, rising from chair, stooping, or pushing a heavy object.

### Statistical analysis

Analyses were performed using SAS, version 9.2 (SAS Institute, Inc., Cary, NC). RAND HRS analytic files, which provided processed data and derived variables [17], were linked to raw questionnaire data. The operational definition of RLS required all three criteria: a) feeling restless, fidgety, or unable to sit still; b) the need to move your legs, rub your legs, or stretch your legs; and c) unpleasant nighttime sensations occurring at least once per week. Crude cross-sectional RLS associations with categorical demographic variables were tested using χ^2^. Ordinal variables were evaluated for trend. Prevalence estimates were weighted for study sampling to reflect the U.S. non-institutionalized population born before 1947, reflecting the underlying HRS cohort.

Potential RLS correlates at baseline were explored by calculating multivariate-adjusted prevalence ratios (PR) derived from Cox proportional hazards regression with robust variance estimates [20]. Dependent variables were modeled with a fixed time effect (uniformly set to 1). Categorical predictors were screened and considered potentially important if the likelihood ratio test was significant at p < 0.20, using forward variable selection and comparisons of the Akaike Information Criteria (AIC). Continuous independent variables were tested in various manners (linear, polynomial, ordinal, or nominal categorical), with selection of best fitting relationship based on AIC. Correlation among predictors was evaluated based on eigenvalues derived from principal component analysis of the predictor variables. Variables were selected so that that each variable contributed uniquely to total variance (i.e., condition indices associated with individual predictor variables were ≤ 30), while maximizing AIC [21]. Additional model-building approaches, such as chunkwise variable selection [22], did not significantly alter the final model.

RLS was then considered as an independent variable, adjusting for significant correlates from the RLS prediction model, to predict concurrent functional limitations. Disability variables were modeled as above to calculate multivariate-adjusted PR. Multi-domain measures (e.g., ADL, IADL) were positive if any component showed limitations. Next, using longitudinal disability data from three subsequent interview waves (2004, 2006, and 2008), RLS was modeled as a predictor of incident functional limitations using Cox proportional hazards regression. Hazard ratios (HR) were reported for each incident disability, limited to the sample at risk (i.e., those without the specific functional limitation in 2002). Censoring occurred at the end of follow-up, death, voluntary withdrawal from the study, or first occurrence study ineligibility (i.e., living in an institutional setting or no longer being married to a spouse in the study cohort). Adjustment for pain and medication use at baseline had the greatest effect on the point estimates. Repeated measures were considered time-varying covariates in the analysis.

## Results

Table 1 reports the baseline characteristics of the sample, with crude tests association. Symptoms of RLS were common (Table 2). The overall prevalence of restless legs syndrome using the operational case definition was 10.6%. RLS was more prevalent in women (p < 0.001), and individual symptoms were more common in women (p < 0.05), with the exception of “twitching or kicking”, which was more frequent in men (p < 0.05). RLS prevalence decreased with increasing age (test for trend, p < 0.05). There were no associations with race (data not shown). Among those with any symptoms, 43.4% discussed their symptoms with a doctor. Of these individuals, 40.4% reported receiving treatment for their symptoms. Women were significantly more likely than men to receive treatment. 78.8% of those who received treatment felt it was successful. Of the treatments mentioned in open-ended questioning (allowing for multiple responses), prescription medication was the most common (26%), followed by exercise / physical therapy / rest (15%), over-the-counter pain medications such as aspirin or ibuprofen (13%), surgery / epidural (7%), topical ointments, heat, or ice (5%), support stockings / ankle weights (4%), vitamins or minerals (4%), change or cessation of current medication (3%), or other medication, not-further specified (27%).

**Table 1:** Characteristics of 2002 Health and Retirement Study restless legs syndrome module (N = 1,008)

**Variable**	**Included (n = 1008)**	(%^*^)	**RLS (n = 104)**	(%^*^)	**No RLS (n = 904)**	(%^*^)
**Age**						
Age 54–64 y	374	46.6	43	52.5	331	45.9
Age 65–79 y	482	40.2	49	37.0	433	40.6
Age 80 y or older	152	13.2	12	10.5	140	13.5
**Gender** ‡						
Male	430	44.4	26	29.2	404	46.2
Female	578	55.6	78	70.8	500	53.8
**Self-reported Race / Ethnicity**						
White, Non-Hispanic	795	84.3	82	86.3	713	84.1
Black, Non-Hispanic	138	8.8	17	8.0	121	8.9
Other	75	6.9	5	5.6	70	7.0
**Education** †						
Less than High School	218	17.4	31	23.2	187	16.7
High School / Equivalent	386	37.8	45	46.0	341	36.8
Some College	205	22.5	17	18.3	188	23.0
College Graduate	199	22.3	11	12.5	188	23.5
**Income** ‡						
Less than $17,600	256	22.5	41	32.7	215	21.3
$17,000 - $33,175	249	21.5	34	30.8	215	20.5
$33,176 - $63,079	259	25.7	15	15.0	244	27.0
$63,080+	244	30.2	14	22.0	230	31.2
**Currently Employed** ‡	354	41.7	20	24.8	334	43.8
**Health Limits Ability to Work** ‡	289	25.8	71	68.8	218	20.8
**Doctor Visits** (In previous 2 y) †						
0-3 Doctor Visits	294	30.6	25	22.1	269	31.6
4-7 Doctor Visits	270	26.4	17	18.9	253	27.3
8-11 Doctor Visits	181	17.5	22	24.9	159	16.7
12 or More Doctor Visits	263	25.4	40	34.1	223	24.4
**Hospitalized** (In previous 2 y) †	260	23.0	36	34.0	224	21.8
**Self-Report of No Medical Plan**	69	7.4	11	11.5	58	6.9
**BMI** †						
< 25.0	345	35.1	25	21.9	336	38.5
25.0-29.9	418	42.0	44	44.0	358	39.9
30.0 or greater	245	23.0	35	34.0	210	21.6
**Smoking Status**						
Never Smoker	403	40.0	38	34.0	365	40.8
Former Smoker	456	45.3	46	49.1	410	44.8
Current Smoker	149	14.7	20	16.9	129	14.4
**Alcohol Consumption** ‡						
Non-drinker	525	48.6	77	70.5	448	46.0
Current Consumption	483	51.4	27	29.6	456	54.0
**Vigorous Activity** (3 times per week) †	427	44.4	34	34.5	393	45.6
**Falls** (In previous 2 y) ‡						
None	824	83.9	74	74.6	750	85.1
Once	103	9.3	12	10.3	91	9.1
2 or More Times	81	6.8	18	15.1	63	5.8
**Self-Reported Health** ‡						
Very Good / Excellent	417	43.8	17	16.0	400	47.1
Good	342	35.3	28	34.3	314	35.4
Fair / Poor	249	20.9	59	49.6	190	17.5
**Self-Reported Pain** ‡						
None	733	73.9	28	29.8	705	79.1
Mild-Moderate	111	10.4	20	16.9	91	9.6
Severe	164	15.7	56	53.3	108	11.3
**Self-Reported Medical Conditions**						
**Hypertension** †	520	48.9	66	60.5	454	47.5
**Diabetes** ‡	164	15.7	32	31.9	132	13.8
**Cancer**	134	12.7	14	10.6	120	13.0
**Lung Disease** ‡	82	7.3	21	17.6	61	6.1
**Heart Disease** ‡	241	21.6	40	38.2	201	19.6
**Stroke**	63	5.2	8	6.9	55	5.0
**Psychiatric Disease** ‡	128	13.2	33	34.1	95	10.7
**Arthritis** ‡	592	54.5	87	83.1	505	51.1
**Self-Reported Chronic Medications** ‡						
None	314	33.8	15	15.0	299	36.1
One Chronic Medication	330	33.5	18	20.4	312	35.0
Two Chronic Medications	214	19.0	26	22.2	188	18.6
Three or More Chronic Medications	150	13.7	45	42.4	105	10.3
**Depressive Symptoms** (CES-D ≥ 2) ‡	219	19.8	57	46.6	162	16.6
**ADL Limitation** (≥ 1 impairment) ‡	136	12.4	40	39.2	96	9.2
**IADL Limitation** (≥ 1 impairment) ‡	97	8.6	34	30.6	63	6.0
**Mobility Limitation** (≥ 1 impairment) ‡	454	41.3	91	87.6	363	35.8
**Large Muscle Limitation** (≥ 1 impairment) ‡	611	56.7	98	95.1	513	52.2
**Gross Motor Limitation** (≥ 1 impairment) ‡	222	20.1	61	56.1	161	15.8
**Fine Motor Limitation** (≥ 1 impairment) ‡	112	10.1	26	28.7	86	7.9

Abbreviations: y = Years; ADL = Activities of Daily Living; IADL = Instrumental Activities of Daily Living; BMI = Body Mass Index; CES-D = Center for Epidemiologic Studies Depression Scale.

* Percentage based on sampling weight to reflect U.S. population aged 54 or older at baseline, reflected in the underlying Health and Retirement Study cohort.

† χ^2^ test for association in all nominal variables and test for trend in all ordinal variables, p < 0.05; ‡ p < 0.001.

**Table 2:** Prevalence* of symptoms related to restless leg syndrome in the 2002 Health and Retirement Study (N = 1,008)

**Question**		**Age Group**			**Gender**	
	**Total**	**50-64**	**65-79**	**80+**	**Female**	**Male**
	**(%)**	**(%)**	**(%)**	**(%)**	**(%)**	**(%)**
**Reported Leg Symptoms at Night**					†	†
Never	59.8	61.3	57.4	62.3	55.1	65.8
Less than once/month	14.0	12.1	16.3	13.7	15.0	12.7
At least once/month but < weekly	6.3	7.4	5.7	4.1	6.6	5.9
At least once/week	19.9	19.2	20.6	20.0	23.3	15.6
**Crawling or aching arms or legs at rest**	37.9	39.8	36.4	35.7	44.0‡	30.2‡
**Restless, fidgety, or unable sit still at rest**	25.2	28.1	23.5	20.2	27.9†	21.7†
**Need to move, rub, or stretch legs at rest**	53.0	53.7	52.0	53.4	57.0‡	47.9‡
**Twitching or kicking of arms or legs**	17.9	21.2†	14.9†	15.2†	15.1†	21.4†
**Itching sensations on body at rest**	20.9	20.2	21.2	22.5	25.2†	15.6†
**Discussed problems with doctor §**	43.4	38.5†	51.1†	39.3†	44.0	42.7
**Prescribed treatment for restlessness ||**	40.4	33.3	43.0	54.1	47.8‡	29.8‡
**Treatment helped ¶**	78.8	81.4	74.9	85.0	80.7	73.9
Case Definition **	10.6	11.9	9.7	8.4	13.5‡	7.0‡

* = Prevalence estimates weighted to represent non-institutionalized U.S. population aged 54 or older.

χ^2^ test for association in all dichotomous questions. Test for trend performed for RLS symptoms at night by gender. For age associations, test for trend was not-significant but χ^2^ test for nominal association was significant. † p < 0.05.

‡ p < 0.001.

**§** Among those with any symptoms (n = 509).

|| = Among those who sought medical advice (n = 239);

**¶** = Among those with prescribed treatment (n = 109).

** = Restless Legs symptoms defined as unpleasant or restless sensations in the legs at least once per month, and reporting “restless, fidgety, or unable to sit still” feelings at rest and the need to “move, rub, or stretch legs” (n = 104).

### Cross-sectional risk factors for RLS

Multivariate regression modeling for potential RLS correlates was performed as described, using forward selection based on AIC improvements. Table 3 shows the full multivariate-adjusted model for factors cross-sectionally associated with RLS. Initially screened predictors of RLS that were not significant in multivariate modeling included race, education, marital status, geographic region, vigorous activity, smoking status, history of hospitalizations, depressive symptoms, or self-reported history of comorbidities (results not shown). The PR for gender was attenuated in the multivariate model. The older age groups were less likely to have RLS. Current alcohol consumption was associated with decreased prevalence of RLS, and modeling alcohol consumption based on frequency and/or amount per session did not improve the model (results not shown). Individuals with other sleep complaints (waking frequently at night or waking early and being unable to return to sleep) were more likely to also have RLS symptoms. Those more affected by bodily pain had significantly higher PR for RLS, as did individuals whose health limits their ability to work, those with frequent falls, those reporting the highest levels of out-of-pocket medical expenses, and those reporting multiple medications for chronic conditions. However, those with more frequent visits to providers had lower PR for RLS after multivariate adjustment. Modeling BMI, total income, and medical expenses as continuous or quadratic functions did not improve the fit (results not shown). There was no evidence of meaningful effect modification in the predictors of RLS.

**Table 3:** Multivariate-adjusted prevalence ratios for factors associated with restless legs syndrome* in the Health and Retirement Study 2002 survey (N = 1008)

**Restless Leg Symptoms Predictor**	**Multivariate Adjusted Model**†		
	**PR**	**95% CI**	**p**
**Age** (reference: 50–64 years)			
Age 65–79 years	0.45	(0.28, 0.72)	<0.001
Age 80 or older	0.52	(0.25, 1.07)	0.07
**Gender** (reference: Male)	1.50	(0.93, 2.40)	0.10
**Income** (reference: Lowest Quartile)			
2nd quartile	1.37	(0.83, 2.27)	0.22
3rd quartile	0.82	(0.46, 1.46)	0.51
4th quartile	1.54	(0.85, 2.81)	0.16
**Current drinking status** (reference: None)	0.59	(0.37, 0.92)	0.02
**Body Mass Index** (reference: BMI < 25.0)			
BMI 25.0 – 29.9	1.77	(1.05, 2.99)	0.03
BMI 30 or greater	1.40	(0.80, 2.46)	0.24
**Pain category** (reference: None)			
Mild to moderate	2.67	(1.47, 4.84)	0.001
Severe	3.44	(2.00, 5.93)	<0.001
**Number of chronic medications used** (reference: None)			
1 chronic medication	1.14	(0.57, 2.26)	0.71
2 chronic medications	1.32	(0.70, 2.46)	0.39
3 or more chronic medications	2.54	(1.26, 5.12)	0.009
**Out of pocket medical expenses** (reference: Highest Quartile)			
2nd quartile	1.65	(0.88, 3.09)	0.12
3rd quartile	1.33	(0.69, 2.57)	0.40
4th quartile	2.12	(1.17, 3.86)	0.01
**Frequency of doctor visits in last 2 years** (reference: 0–3 visits)			
4-7 visits	0.89	(0.43, 1.82)	0.74
8-11 visits	0.82	(0.46, 1.46)	0.50
12 or more visits	0.49	(0.27, 0.90)	0.02
**Health limits ability to work**	2.91	(1.75, 4.85)	<0.001
**Falls in the last 2 years** (reference: None)			
Fell once	1.33	(0.73, 2.40)	0.35
Fell 2 or more times	2.63	(1.49, 4.66)	<0.001
**Associated sleep problems**			
**Problems with early waking**	1.69	(1.09, 2.62)	0.02
**Problems with frequent waking**	1.55	(1.00, 2.41)	0.05

Abbreviations: PR = prevalence ratio; CI = confidence interval; BMI = Body Mass Index.

* Restless Legs symptoms defined as unpleasant or restless sensations in the legs at least once per week, and reporting “restless, fidgety, or unable to sit still” feelings at rest and the need to “move, rub, or stretch legs” (n = 104).

† Final multivariate model reflecting automated variable forward selection with improvements to Akaike Information Criterion statistic.

### RLS and concurrent disability

Individuals with RLS were more likely to have certain functional status limitations at baseline (Table 4, adjusted for correlates reported in Table 3; specifically age, gender, moderate/severe pain, drinking status, BMI, health limiting ability to work, previous falls, other sleep symptoms, income, out-of-pocket medical expenses, frequency of provider visits, and number of chronic medications). RLS was not associated with significant PR (p > 0.20, not shown) for several individual outcomes, including: difficulties walking short distances (across the room, one block, or requiring assistance to walk); difficulty climbing one flight of stairs; difficulties picking up a dime or carrying 10 lb; certain specific ADL limitations (feeding oneself); and most IADL limitations (reading a map, managing money, managing medications, or shopping for groceries). RLS sufferers have elevated PR for several aggregate functional outcomes (i.e., mobility limitations, large muscle group limitations, ADL, and IADL) but not aggregate gross motor and fine motor variables (Table 4).

**Table 4:** Cross-sectional associations (Prevalence Ratios) between restless legs syndrome* and functional limitations in the Health and Retirement Study 2002 survey (N = 1008)

**Functional Limitation Outcome**	**Total (N)**	**Limited (N)**	**%**	PR*	**95% CI**	**P**
**Mobility limitations (At least 1)**	1008	454	45.0	1.25	(1.08, 1.46)	0.004
Some difficulty walking several blocks	994	273	27.5	1.43	(1.12, 1.82)	0.004
Difficulty climbing several flights	900	408	45.3	1.16	(0.98, 1.36)	0.09
**Large muscle group limitations (At least 1)**	1008	611	60.6	1.20	(1.06, 1.36)	0.004
Some difficulty sitting for 2 hours	997	191	19.2	1.29	(0.95, 1.76)	0.11
Some difficulty rising from chair	1007	397	39.4	1.20	(0.96, 1.49)	0.11
Difficulty with stooping	997	451	45.2	1.15	(0.96, 1.38)	0.14
Pushing or pulling a large object	957	227	23.7	1.56	(1.18, 2.07)	0.002
**Gross motor limitations† (At least 1)**	1008	222	22.0	1.13	(0.86, 1.49)	0.37
**Fine motor limitations‡ (At least 1)**	1008	112	11.1	1.42	(0.89, 2.26)	0.14
**Difficulty reaching or extending arms up**	1007	139	13.8	1.68	(1.10, 2.55)	0.02
**ADL limitations (At least 1)**	1008	136	13.5	1.46	(1.00, 2.11)	0.05
**Specific ADLs**						
Dressing oneself	1008	74	7.3	2.23	(1.26, 3.95)	0.006
Bathing oneself	1008	53	5.3	1.69	(0.80, 3.59)	0.17
Transferring into or out of bed	1007	46	4.6	2.07	(1.01, 4.23)	0.05
Toileting	1008	51	5.1	2.16	(1.04, 4.52)	0.04
**IADL limitations (At least 1)**	1008	97	9.6	1.71	(1.13, 2.59)	0.01
**Specific IADLs**						
Using a telephone	1008	20	2.0	0.26	(0.06, 1.22)	0.09
Preparing Meals	952	43	4.5	2.96	(1.47, 5.95)	0.002

* Restless Legs symptoms defined as unpleasant or restless sensations in the legs at least once per week, and reporting “restless, fidgety, or unable to sit still” feelings at rest and the need to “move, rub, or stretch legs” (n = 104). Adjusted for significant covariates from analysis in Table 3, including: age, gender, income category (quartiles), health interfering with ability to work, pain symptoms (mild-moderate or severe compared to none), other sleep symptoms (walking too easily or too early), body mass index, current drinking status, number of chronic medications used, out-of-pocket medical expenses (quartiles), number of doctor visits in the last 2 y, and falls in the previous 2 y.

† Gross motor limitations defined as at least some difficulty in one of these domains: Walking 1 block, walking with an assistive device, climbing 1 flight of stairs, transferring, or bathing.

‡ Fine motor limitations defined as at least some difficulty in one of these domains: Picking up a dime, feeding oneself, or dressing oneself.

Abbreviations: PR = prevalence ratio; CI = confidence interval; ADL = Activities of Daily Living; IADL = Instrumental Activities of Daily Living; y = years.

### RLS and incident disability

Next, we examined hazard ratios (HR) for incident functional limitations between RLS sufferers and non-sufferers for each disability outcome variable during the following three survey waves. Crude associations between RLS and incident disability ranged from roughly two- to four-fold increased rates (Table 5). However, with adjustment for baseline covariates (similar to the cross-sectional analysis from Table 3, although allowing for time-dependent changes during follow-up), RLS did not predict significant increases for incident limitations of the aggregate measures (i.e, mobility limitations, ADL, IADL, gross and fine motor limitations, or large muscle groups), although mobility limitations approached statistical and clinical significance (HR: 1.90, 95% confidence interval (0.97, 3.70), p = 0.06). RLS did predict increases in some specific functions, including difficulties: climbing several stair flights; raising from a chair; sitting prolonged periods; stooping or kneeling; pushing or pulling large objects; picking up a dime; lifting 10 lb; or reaching arms up). Again, no limitation was seen for walking short distances. No specific ADL or IADL functions were significantly different for RLS sufferers during follow-up.

**Table 5:** Incident functional limitations predicted by Restless Legs Syndrome* at baseline (2002, N = 1008) in the Health and Retirement Study (Follow-up until 2008)

**Functional Limitation Outcome**			**Unadjusted**†			**Multivariate Adjusted**†		
	**At Risk**	**Disability**	**HR**	**95% CI**	**p**	**HR**	**95% CI**	**p**
**Mobility limitations**	524	247	2.26	(1.21, 4.23)	0.01	1.90	(0.97, 3.70)	0.06
**Difficulty climbing several flights**	564	246	3.13	(2.03, 4.85)	<0.001	2.38	(1.39, 4.06)	0.002
**Difficulty climbing one flight**	780	180	3.62	(2.36, 5.55)	<0.001	1.51	(0.96, 2.40)	0.08
**Large muscle group limitations**	371	210	1.97	(0.86, 4.53)	0.11	1.01	(0.48, 2.12)	0.98
**Some difficulty sitting for 2 hours**	745	178	3.26	(2.12, 5.01)	<0.001	2.17	(1.25, 3.75)	0.006
**Some difficulty rising from chair**	565	224	3.16	(2.11, 4.73)	<0.001	2.54	(1.62, 3.99)	<0.001
**Difficulty with stooping**	518	245	4.09	(2.86, 5.86)	<0.001	2.66	(1.71, 4.15)	<0.001
**Pushing or pulling a large object**	721	238	3.23	(2.01, 5.19)	<0.001	1.79	(1.08, 2.99)	0.02
**Gross motor limitations† (At least 1)**	731	212	2.43	(1.46, 4.03)	0.001	0.91	(0.52, 1.59)	0.73
**Fine motor limitations‡ (At least 1)**	826	154	2.45	(1.50, 4.01)	<0.001	1.18	(0.72, 1.94)	0.52
**Difficulty with lifting or carrying 10 lb**	744	206	3.02	(1.99, 4.59)	<0.001	1.61	(1.05, 2.46)	0.03
**Difficulty picking up a dime**	872	89	2.97	(1.69, 5.21)	<0.001	1.97	(1.12, 3.46)	0.02
**Difficulty reaching or extending arms up**	799	173	3.22	(2.06, 5.06)	<0.001	1.76	(1.05, 2.97)	0.03
**ADL limitations (At least 1)**	807	162	1.94	(1.11, 3.40)	0.02	0.64	(0.36, 1.13)	0.12
**IADL limitations (At least 1)**	843	139	2.62	(1.50, 4.57)	0.001	1.05	(0.60, 1.82)	0.87

* Restless Legs symptoms defined as unpleasant or restless sensations in the legs at least once per week, and reporting “restless, fidgety, or unable to sit still” feelings at rest and the need to “move, rub, or stretch legs” (n = 104 in 2002).

† Unadjusted hazard ratio shows incident disability. Functional limitation was assessed biannually, subjects censored if no longer eligible at follow-up waves. Multivariate-adjusted hazard ratio includes variables adjusted for in Table 3 (cross-sectionally associated with RLS, including: age, gender, income category (quartiles), health interfering with ability to work, pain symptoms (mild-moderate or severe compared to none), other sleep symptoms (walking too easily or too early), body mass index, current drinking status, number of chronic medications used, out-of-pocket medical expenses (quartiles), number of doctor visits in the last 2 y, and falls in the previous 2 y), as well as time-varying covariates associated with RLS in the follow-up waves (incident stroke, incident psychiatric diagnosis).

† Gross motor limitations defined as at least some difficulty in one of these domains: Walking 1 block, walking with an assistive device, climbing 1 flight of stairs, transferring, or bathing.

‡ Fine motor limitations defined as at least some difficulty in one of these domains: Picking up a dime, feeding oneself, or dressing oneself.

Abbreviations: HR = hazard ratio; CI = confidence interval; ADL = Activities of Daily Living; IADL = Instrumental Activities of Daily Living; y = years; lb = pounds.

## Discussion

This population-based sample of U.S. elders confirms that restless legs syndrome is common, with 10.6% of our sample meeting the operational definition of weekly RLS symptoms, and potentially disabling. The prevalence estimates are consistent with other reports, although we were unable to classify patients with primary versus secondary RLS. The multinational RLS Epidemiology, Symptoms, and Treatment (REST) study found annual period prevalence of 7.2%, although 5% had weekly symptoms [5]. The 2005 National Sleep Foundation Poll’s estimate was 9.7% for symptoms several nights per week [4]. The MEMO study, another population-based survey, reported 9.8% prevalence in elders in Germany [7]. Secondary RLS can be very common, such as reports of 20% in hemodialysis patients [10], although primary RLS is less common, estimated at 2.4% in the U.S. [9].

Among those reporting RLS symptoms, fewer than half sought medical care. Among those who discuss their symptoms, fewer than half received treatment. However, most who received treatment reported symptom improvement. This pattern confirms reports from the REST study, suggesting delays in both diagnosis of RLS and initiation of appropriate therapies [5]. The literature suggests RLS patients have good response to therapy in 90% of patients when correctly diagnosed [2]. Evidence-based guidelines for RLS treatment now exist [23], although one clinic-based German study found no clinical improvement with evidence-based guideline adherence [24]. In the REST study, 81% of RLS patients with distressing symptoms discussed their symptoms with their physicians, but only 24% of these were given any diagnosis [3,5]. The majority of patients with RLS were diagnosed with circulation problems, arthritis, back or spine problems, varicose veins, depression, anxiety, or trapped nerves, but only 6% were correctly diagnosed with RLS [5].

This study identifies associations between RLS and disability using valid functional limitation measures. RLS sufferers were 46% more likely to have ADL limitations at baseline, adjusted for covariates, and several ADL domains had doubled risk (dressing oneself, transferring, and toileting). RLS sufferers were also 71% more likely to have IADL limitations, including nearly three-fold increase in difficulty making meals by themselves. RLS sufferers were more likely to have concurrent mobility limitations (25% increase) and limitations requiring large muscle groups (20% increase), but did not have significant changes in fine or gross motor functions. However, some specific limitations, such as lifting their arms, pushing heavy weights, and rising from a chair, were significantly increased. Associations with mobility measures were more prominent in more strenuous tasks (multiple blocks or multiple stair flights).

This study is the first population-based study to longitudinally assess functional measures among adults with RLS. Despite adjustment for all baseline and time-dependent covariates, specific functional limitations were significantly increased among subjects with RLS. RLS suffers were more likely to develop specific incident disabilities, which involving the core, lower extremities, and upper extremities, such as difficulty climbing stairs (HR = 2.38), difficulty stooping (HR = 2.66), difficulty rising from a chair (HR = 2.54), difficulty sitting for 2 hours (HR = 2.17), moving large objects (HR = 1.79), raising arms (HR = 1.76), or picking up a dime (HR = 1.97). However, no aggregate measures (ADL, IADL, gross motor, fine motor, mobility, or large muscle group limitations) reached statistical significance during follow-up, after adjustment for baseline characteristics (particularly pain and chronic medication use). If pain mediates how RLS affects disability, this may reflect over-adjustment. Also, excluding disabled patients at baseline markedly decreased the population at-risk for new disability, limiting the power of the study. We did not model transitions from mild to more severe disability.

The difficulty with prolonged sitting follows with the expected perturbation of RLS symptoms during periods of inactivity. Sleep symptoms (frequent waking or waking early without being able to fall asleep) were significantly associated with RLS. We adjusted for these sleep symptoms when analyzing the potential impact of RLS on disability, although symptoms interfering with sleep quality may be an intermediate factors between RLS and disability. Multiple studies have considered role limitations and quality of life. RLS patients have been shown to have decreased scores on the Medical Outcomes Study 36-Item Short Form (SF-36) in all tested domains [5,15]. The National Sleep Foundation (NSF) poll noted that RLS sufferers were more likely to be unemployed and to have difficulties at work [4]. We confirmed that employed elders outside of the home were less likely to have RLS (age- and sex- adjusted PR 0.38, 95% CI 0.21-0.68, although not significant in multivariate analysis), and those who report that there health limited their ability to work were three times more likely to have RLS. The REST study reported that RLS sufferers reported daytime sleepiness (32%), difficulty concentrating (15%), and negative influences on daily activities and personal and work life [5]. The NSF poll also found evidence of daytime symptoms in patients with RLS [4]. These findings were replicated in a geriatric sample, with poor sleep quality, daytime somnolence, and low social functioning due to impaired tolerance for inactivity [16]. One Scandinavian study showed similar impact on sleep quality, but noted lower SF-12 physical domain scores but not mental domain scores [6]. An analysis of health-related quality of life (HRQOL) in RLS patients in multiple sites in Germany suggested that sleep deficits, duration of symptoms, and household income mediated RLS associates with clinical rating scales, with RLS effects consistent with other chronic neurologic diseases [13]. A Swedish study, limited to women, suggested statistically and clinically significant deviations in HRQOL although mental domains predominated [14]. When HRQOL was assessed in the REST study, McKrink et al. were able to estimate factors related to RLS diagnosis, severity, and treatment using SF-36 as predictors of the domains, but no comparison to patients without RLS was possible [12]. One recent small study of RLS patients showed substantial cognitive deficits similar to those deprived of sleep for one night, despite assessment at a time when RLS symptoms were not active [25]. We did not find concomitant cognitive dysfunction associated with RLS (results not shown).

Our risk factor analysis yielded results consistent with other studies. Women were nearly twice as likely to have RLS based on age-adjusted estimates; however, this difference was reduced by half when adjusted for other covariates, such as pain syndromes, alcohol consumption, BMI, age, medication use, and health care utilization, suggesting that gender differences in RLS prevalence may be partially due to confounding. There was no evidence of significant effect modification by gender, although there was a relatively small number of males with RLS (n = 26). Although RLS is thought to be more common in the elderly, the prevalence of RLS trended downward with age in our sample. Several studies report increased prevalence with age, peaking around age 65 [4,5], consistent with our reference group, which was those aged 50–64.

RLS is known to have a strong hereditary component [8] but may be associated with acquired factors such as increased BMI, inactivity, and low alcohol consumption [26]. Vigorous activity was crudely associated with RLS but the association did not persist in multivariate modeling. We observed that those with some alcohol consumption tended to have decreased prevalence of RLS, similar to Phillips and colleagues [26], despite modeling alcohol consumption in various ways. There were no independent effects for “binge drinking”, frequency of drinking, or amount per session. Cigarette smoking associations have been inconsistent [4,6,7,26,27] and smoking status was not associated with RLS in this sample. Crude associations with arthritis, hypertension, diabetes, lung disease, and heart disease were observed, but adjustment for general health status and symptomatic variables were better predictors of RLS cross-sectionally. Although RLS may be secondary to conditions like COPD [6], anemia, kidney disease, and diabetes, we did not observe persistent effects for medical comorbidities in this study. Psychiatric comorbidity or depressive symptoms also did not predict RLS after adjustment, despite reports that mood disorders are more common in RLS sufferers [7,28-30]. We did adjust for medication use and sleep-related symptoms, which may obscure this association. Similarity, self-reported health status had previously been noted as a predictor of RLS [26], but did not retain significance in our study. RLS is common in patients with fibromyalgia [31,32], and sleep quality can be a determinant of HRQOL in these patients. Heavy medication use was associated with RLS, although we could not assess if this was causal or merely an indicator of multiple comorbidities.

Pain experience was a strong predictor of RLS, as was history of frequent falls. There may be overlap between RLS and chronic neuropathic pain pathophysiology, as both are mediated by the dopaminergic system [33]. Neuropathic pain treatments are effective on RLS symptoms [33], and non-opioid analgesic use predicted RLS among patients taking tricyclic and serotonin-reuptake-inhibiting antidepressants [34]. Most RLS sufferers in that study had chronic recurrent pain, and nearly all used analgesics [35]. RLS patients are more sensitive to pain, measured by pinprick hyperalgesia in the extremities [36]. Furthermore, RLS patients showed worse bodily pain scores using SF-36 [15]. Pain is not recognized in the standardized definition of RLS, but if pain is part of the clinical syndrome, increased pain may, at least partially, explain RLS associations with incident functional limitations. Our findings support the hypothesis that RLS sufferers may suffer from other chronic pain syndromes. Or, patients with neuropathic pain syndromes may some symptoms that mimic RLS [9].

There are limitations to this work. First, the 2002 RLS module was part of the pre-existing HRS cohort and not a principle aim for the study, and data on RLS was collected at one time point and was without clinical evaluation. Risk factors and correlates, including functional limitations, were available both before and after this assessment. We did not differentiate between primary and secondary RLS. Standardized diagnostic criteria suggested by the International RLS Study Group [2] were not strictly applied, but our case definition included the essence of the core diagnostic criteria at least weekly. Our case definition did not require the characteristics of movement providing symptom alleviation or that the symptoms worsen at night, which are considered diagnostic criteria for RLS. However, the questions did specify that the symptoms were present at rest. Our prevalence was consistent with other studies in elders, but extrapolation of our findings should be limited to those aged 54 or older. Although unmeasured confounding is always a concern, we also risk over-adjustment in multivariate modeling. Some crude estimates were markedly changed with control for pain and medication use. We do not know if pain is a mediating variable or part of the clinical syndrome rather than a true confounder. We also did not confirm chronic diseases as covariates with RLS as seen in other studies, but our general markers of health status and medication use better fit the data. We also could not assess for specific medication class effects, such as medications that cause akathisia (i.e., anti-psychotics and anti-depressants) [37], which has clinical similarities to RLS. Some of the RLS treatments reported by subjects (e.g., “surgery or epidural”) could be potentially disabling. Although we could detect incident functional limitations, we excluded subjects with specific disabilities at baseline. For the most common disabilities, this reduced the sample size considered at risk, which also decreased the power to detect differences. Furthermore, as RLS was only assessed at one time-point, associations that may be “early” or “late” manifestations of disability would be differentially detected.

## Conclusions

In this study, we have corroborated reports that RLS may be substantially under-diagnosed and undertreated during this period. RLS predicted functional decline in multiple domains, which suggests an impact on subjects’ abilities to work and live independently. If the observed limitations are reversible when appropriate treatment is given, early detection and treatment of RLS may help remediate RLS functional decline.

## Competing interests

The authors declare that they have no competing interests.

## Authors’ contributions

DJC conducted the statistical analyses and drafted the manuscript. Both authors were involved in the conception of the research and interpretation of the data. RBW is a co-principle investigator for HRS, has ongoing involvement in the design and maintenance of the cohort, and was responsible for devising the survey questions use in this manuscript. Both authors read and approved of the final manuscript.

## Pre-publication history

The pre-publication history for this paper can be accessed here:

http://www.biomedcentral.com/1471-2318/12/39/prepub
